# Social media facilitates disclosure among people experiencing child maltreatment: A brief report

**DOI:** 10.1016/j.chipro.2025.100158

**Published:** 2025-04-24

**Authors:** Laura M. Schwab-Reese, Morgan E. PettyJohn, Rafia Tasnim, Michelle Fingerman

**Affiliations:** a Department of Public Health, Purdue University, USA; b School of Social Work, The University of Texas at Arlington, USA; c Independent Scholar, USA

**Keywords:** Child abuse, Child maltreatment, Disclosure, Social media, Online

## Abstract

**Background:**

Many victims, particularly adolescents, must disclose abuse or neglect to have their experiences identified by people able to provide support and resources. Social media may be part of how young people seek support.

**Objective:**

This brief reports the percentage of young victims of maltreatment who use social media to disclose their experiences and describes the most used platforms.

**Participants, setting, and methods:**

Of the 641 individuals who completed a screening survey via Connect on CloudResearch, 111 experienced childhood maltreatment and shared their experiences on social media. Of these, 26 participants completed a follow-up survey.

**Findings:**

Almost all respondents in the follow-up survey reported offline disclosure (n = 24; 92 %), although telling an adult offline was less common (n = 19; 73 %). Most participants disclosed maltreatment on multiple platforms (n = 22; 85 %), usually on two or three platforms (n = 14; 54 %). Although it was common to use anonymous accounts to talk about maltreatment, 80 % of participants posted at least once on their personal accounts.

**Conclusion:**

This study identifies the importance of social media for young people’s maltreatment disclosures. Social media platforms and users need to be prepared to respond appropriately.

## 1. Introduction

More than 7.5 million children and adolescents are reported to Child Protective Services (CPS) each year in the U.S. (U.S. Department of Health & Human Services, Administration for Children and Families, 2022), which likely underestimates the true burden of child maltreatment. Many victims, particularly adolescents, must disclose abuse or neglect to receive help. Some of the negative consequences of maltreatment, such as worsened physical, mental, social, and economic well-being (Hughes et al., 2017), can be reduced by connecting young people with support and resources (Kogan, 2004). Social media may be part of how young people seek support (Schwab-Reese et al., 2019; Williams et al., 2023); however, it is unclear how many young people use social media to seek child maltreatment-related support or the preferred platforms. The goal of this research brief is to report the percentage of young victims of maltreatment using social media to disclose these experiences and to describe the most commonly used platforms.

## 2. Methods

From December 2023 through February 2024, we deployed a screening survey via Connect on CloudResearch (Hartman et al.) to 661 individuals, asking about experiences of child maltreatment and related disclosures on social media. Eligibility criteria included: ages 18–21 years (15 excluded); U.S. resident (3 excluded); and English proficiency (2 excluded). We asked about the current season in the U.S. to screen out respondents who provided low-quality answers (3 excluded). In total, we excluded 20 respondents (final sample: 641).

**Fig. 1.**
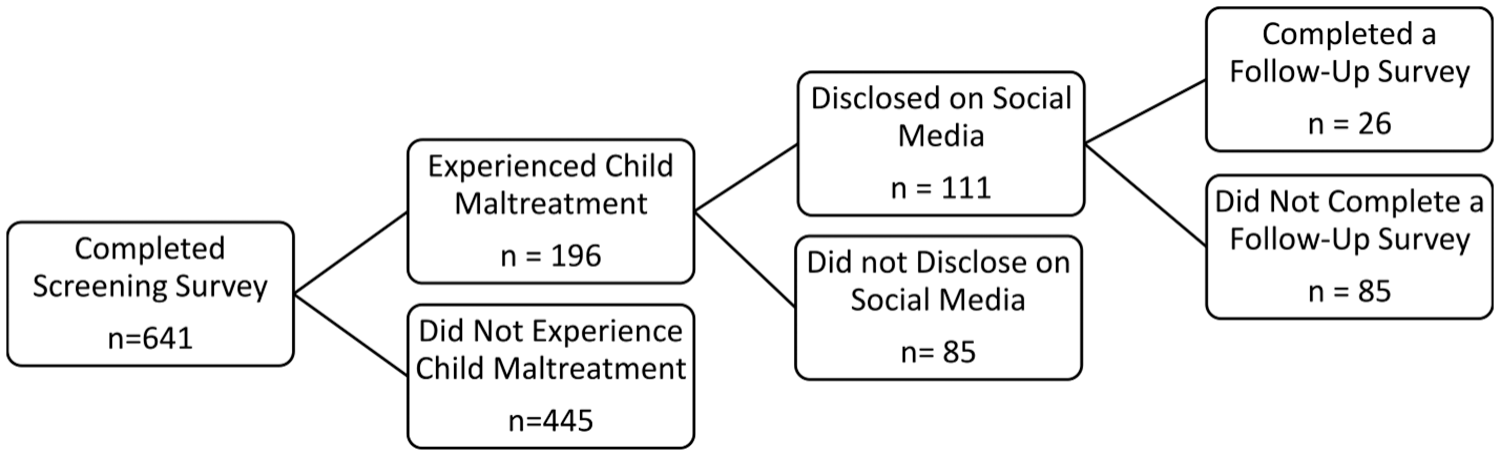
Path from screening survey to follow-up survey.

Of this group, 111 participants (17.2 %) endorsed both experiencing child maltreatment and disclosing these experiences on social media (Fig. 1). Of the 111 who used social media to disclose their maltreatment experiences, 96 provided a valid email address and received a follow-up survey, which explored these topics in greater detail. Twenty-six individuals completed the follow-up survey (response rate: 27.1 %). The second author’s university institutional review board approved the study as minimal risk. Before completing the follow-up survey, all individuals consented to participate in the study.

## 3. Results

More than 30 % of screening survey respondents (n = 196/641) reported that they “experienced abuse or neglect during childhood.” Of these, more than half (n = 111/196; 56.6 %) reported that they “posted about or discussed their experiences with childhood abuse/neglect on any social media platform.”

The follow-up survey sample (n = 26) was demographically diverse. About half identified as cisgender women (n = 14), while others identified as non-binary (n = 5), cisgender men (n = 4), and transgender individuals (n = 3). Respondents could select multiple racial/ethnic identities. The most common were Caucasian/White (n = 14), Hispanic/Latinx (n = 7), African American/Black (n = 6), and Asian American/Asian (n = 6); one participant identified as Native American/Alaska Native. Nearly all had graduated from high school (n = 24), and most were either enrolled in post-secondary education (n = 18) or had completed a college degree (n = 4). Participants reported an average of 6.5 adverse childhood experiences (ACEs; SD = 2.1). The most frequently reported ACE was psychological maltreatment (n = 24), followed by physical maltreatment (n = 22) and witnessing intimate partner violence between caregivers (n = 22).

Because the survey focused on how and where people disclosed maltreatment on social media, all respondents in the follow-up survey sample reported disclosing maltreatment online. A strong majority also shared their maltreatment experiences with someone in an offline setting (92 %; Table 1). It was also common, although relatively less so, to share the experience with an adult, such as a parent, teacher, or counselor (73 %).

In their online disclosures, respondents reported using an average of 3.2 (SD = 1.3) different disclosure approaches (Table 1). Direct messaging was the most common (n = 22; 85 %), but all disclosure methods were used by more than half of the participants. Many (42 %) used a combination of both personal (identifiable) and anonymous social media accounts when making disclosures.

**Fig. 2.**
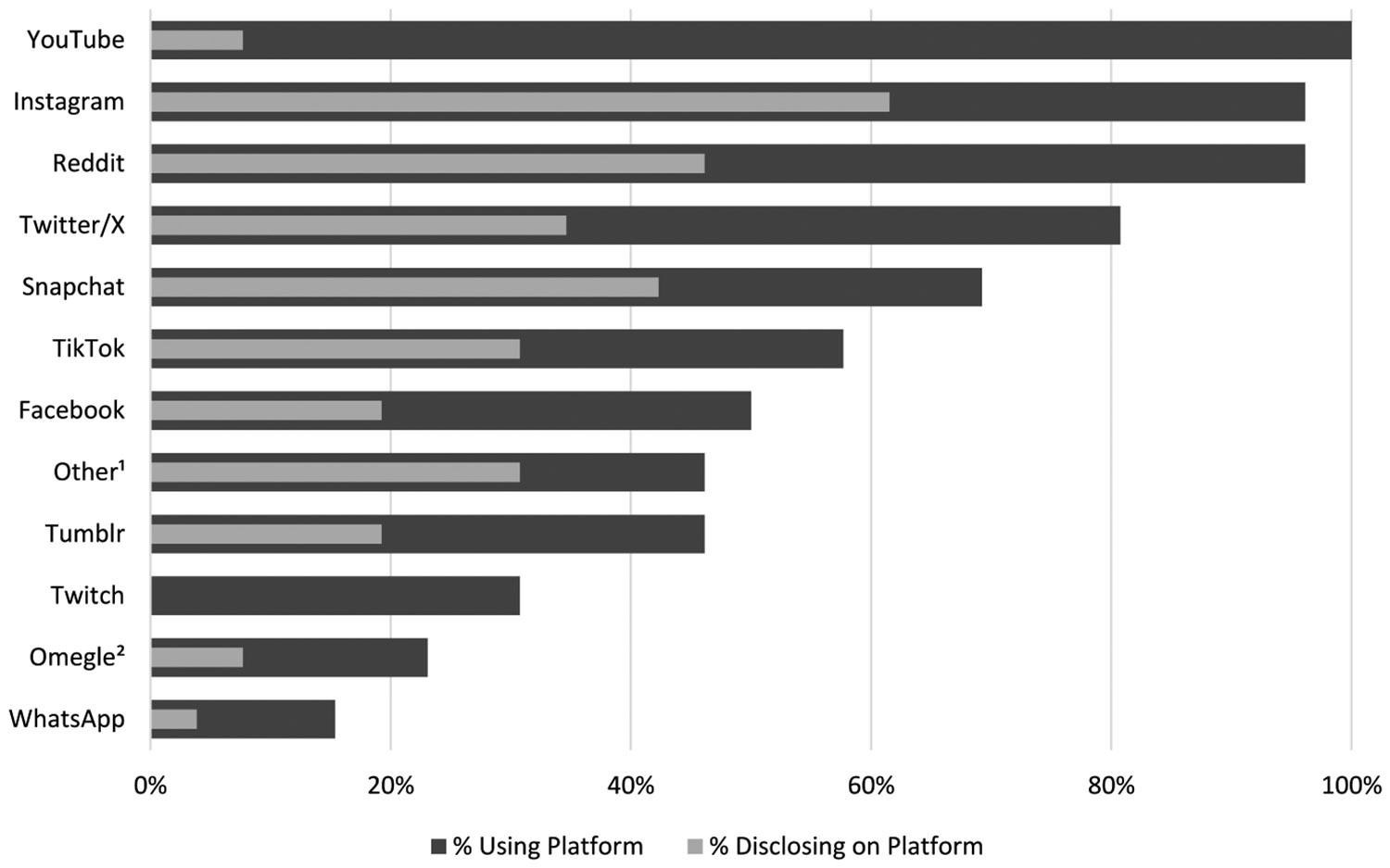
Percent of respondents using social media platforms and disclosing maltreatment on the platform (n = 26). ^1^Includes Discord, Skype, Roblox, TalkLife, Pinterest, Kik, Vine, Steam, Trevor Space ^2^Omegle closed in 2023 due to safety concerns

Regarding general social media use, all follow-up respondents reported using YouTube, and nearly all used Instagram and Reddit (n = 25; Fig. 2). Other frequently used platforms included Twitter/X (n = 21), Snapchat (n = 18), and TikTok (n = 15). On average, participants reported using 7.2 platforms (SD = 2.3). However, different patterns emerged when asked specifically about disclosing child maltreatment. For example, although all participants used YouTube, only two (8 %) disclosed maltreatment there. In contrast, nearly all participants (93 %; n = 25) used Instagram, and among those, almost two-thirds (n = 16) reported disclosing maltreatment on that platform. In addition, 12 participants (46 %) used platforms not included in the main list (e.g., Discord, Skype, Roblox, TalkLife), and 67 % of those individuals used one of these alternative platforms to discuss maltreatment.

## 4. Discussion

In our sample, more than 50 % of people who experienced child maltreatment discussed these experiences on social media. Adding to a growing body of research (Bogen et al., 2021; Lev-Wiesel & First, 2018; Schwab-Reese et al., 2019; Williams et al., 2023), this work demonstrates that the internet, and social media in particular, provides opportunities for young people who choose to disclose maltreatment.

Interestingly, the platforms used most frequently for disclosure were not always the most used overall. Some popular platforms, like Instagram, Reddit, Snapchat, and TikTok, were also used to discuss maltreatment. Less mainstream platforms, such as Discord, Roblox, TalkLife, and Trevor Space, were also used frequently (Gottfried). In contrast, some other widely used platforms, like YouTube, were rarely used for disclosure. Some platforms likely have features that appeal to young people disclosing child maltreatment. Our study was unable to determine how decisions were made regarding when and where to disclose maltreatment. As a result, it is not clear why these platforms were most commonly used, and future research is needed to understand how young people make these decisions.

We also found that young people disclose their child maltreatment experiences on multiple platforms. Disclosure on multiple platforms may suggest that needs, such as support, resources, or information, were not met, prompting additional disclosures. This finding underscores the importance of ensuring that social media platforms and users are prepared to respond in affirming, trauma-informed, and supportive ways. For example, platforms may be able to adopt automated processes that direct individuals to relevant resources, similar to Google’s response to violence. For a range of violence-related search terms (e.g., I’m being raped/beaten/abused), the first item returned is contact information for a relevant hotline. Other platforms may be adopting similar approaches, although we could not find documentation of these efforts. Social media platforms could also provide educational resources and referrals within their own help, resource, or safety centers.

Although resources are needed for individuals experiencing maltreatment, other users may also benefit. These users may be well positioned to respond to disclosures of child maltreatment by offering support or suggesting appropriate resources. Prior research suggests that users on mental health-focused platforms frequently offer high-quality advice, emotional support, and other positive responses (Williams et al., 2023). With appropriate tools and education, this approach could be expanded to more general social media platforms. Educational content may include guidance on recognizing indicators of child maltreatment, strategies for providing supportive responses to disclosures, and information about offline safety resources. Such materials could be made available through resource or safety centers on the platforms; however, they may also be integrated more seamlessly into user experiences through brief pop-up messages or in-app prompts, thereby reducing the need for active user engagement. Regardless of the format, any interventions should be rigorously evaluated before implementation to ensure effectiveness and minimize unintended consequences.

Our study has limitations that should be addressed in future research. First, our sample may not be representative of the overall experiences of young people. We used an online survey platform that would not be accessible to young people without internet access. Further, the sample of individuals willing to discuss their experiences with a researcher may not represent all experiences. Second, our screening was based on individual self-report of maltreatment and disclosure. Due to limited space in the screening, we asked a single dichotomous question about experiences before age 18 with abuse and neglect. As a result, it captured only individuals who conceptualized their experiences as maltreatment, which is likely a different group than those identified through formal systems or who experienced abusive or neglectful behaviors without recognizing them as maltreatment. Our question about social media disclosures was similarly brief, and asking more detailed questions about platforms or disclosure behavior may have resulted in different responses.

In sum, our findings highlight the role of social media in young people’s disclosure of child maltreatment. These disclosures represent both a need and an opportunity: a need for safe and supportive spaces and an opportunity to reach young people with innovative and scalable interventions. Supporting young people who disclose, as well as those who receive these disclosures, will be essential to improving short- and long-term outcomes for youth affected by maltreatment.

## Figures and Tables

**Table 1:** Characteristics of maltreatment disclosure (n = 26).

	n (percent)
Disclosed Offline	
Yes	24 (92 %)
No	2 (8 %)
Disclosed to an Adult Offline	
Yes	19 (73 %)
No	7 (27 %)
Approach to Social Media Disclosure	
Post to Public Forum^a^	16 (62 %)
Post to Private Forum^b^	18 (69 %)
Direct Messaging^c^	22 (85 %)
Sharing Others’ Posts	14 (54 %)
Comments on Others’ Posts	14 (54 %)
Type of Social Media Account(s) Used	
Anonymous Account	5 (19 %)
Personal Account	10 (39 %)
Both Types	11 (42 %)

a Content that could be viewed by anyone (e.g., tweet, Facebook status update).

b Content shared with a restricted group of people (e.g., limited friend lists, membership only boards).

c Private, one-on-one communications.
